# 701. An Open-label Phase 2a Study of Ibezapolstat, a Unique Gram-positive Selective Spectrum (GPSS) Antibiotic, for Patients with *Clostridioides difficile* Infection

**DOI:** 10.1093/ofid/ofab466.898

**Published:** 2021-12-04

**Authors:** Kevin W Garey, Khurshida Begum, Chenlin Hu, Weiqun Wang, Chris Lancaster, Anne J Gonzales-Luna, Caroline Loveall, M Jahangir Alam, Michael Silverman

**Affiliations:** 1 University of Houston College of Pharmacy, Houston, Texas; 2 University of Houston, Houston, Texas; 3 University of Houston, College of Pharmacy, Houston, TX; 4 Acurx Pharmaceuticals, LLC, White Plains, NY

## Abstract

**Background:**

Ibezapolstat, a DNA polymerase IIIC inhibitor, currently in Phase 2 clinical development for treatment of *C. difficile* infection (CDI). Its unique mechanism of action targets low G+C content Gram-positive bacteria primarily Firmicutes including *C. difficile*. Phase I healthy volunteer results demonstrated a favorable microbiome profile suggestive of an anti-recurrence effect. The purpose of this study was to report clinical outcomes, pharmacokinetics, and microbiome changes from this Phase 2a clinical study and to continue to test for anti-recurrence microbiome properties.

**Methods:**

Ibezapolstat 450 mg was given twice daily for 10 days to patients with mild-moderate CDI defined as diarrhea plus a positive *C. difficile* toxin test. Test of cure was evaluated at day 12 and sustained clinical cure at day 38. Stool samples were evaluated for *C. difficile* cultures and microbiome changes.

**Results:**

Ten subjects (female: 50%) aged 50 ±15 years were enrolled. All ten subjects experienced a clinical cure by the test of cure visit at day 12 and all 10 subjects experienced a sustained clinical cure at the day 38 visit. Ibezapolstat was well tolerated with 1 adverse event (nausea) probably related to drug. Ibezapolstat systemic exposure was minimal with no plasma level reaching 1 ug/mL any time during therapy. Ibezapolstat colonic concentrations averaged 400 ug/g stool at day 3 and greater than 1,000 ug/g by day 10 of dosing. Six of the seven available baseline stool samples grew toxigenic *C. difficile* of various ribotypes including RT078-226 and RT014-020 (Ibezapolstat MIC range: 0.25-1 ug/mL). Follow-up cultures were no growth starting from day 3 stool cultures. Microbiome changes included overgrowth of Actinobacteria and/or Firmicute phylum species while on therapy.

**Conclusion:**

Favorable clinical efficacy and safety results were observed in ibezapolstat patients with CDI including 100% clinical cure and sustained clinical cure. These results begin to validate our approach to ibezapolstat development in that the favorable microbiome effects seen in healthy Phase 1 volunteers may be predictive of beneficial patient outcomes, including low rates of recurrence. These results support the continued clinical development of ibezapolstat.

**Disclosures:**

**Kevin W. Garey, Pharm.D., M.S., FASHP**, **Summit Therapeutics** (Research Grant or Support) **Michael Silverman, MD**, **Acurx Pharmaceuticals** (Consultant)

